# Clinical Prognostic Factors and Survival Risk Stratification for Advanced Biliary Tract Cancer Treated with Gemcitabine-Based Palliative Chemotherapy: A Real-World Retrospective Study

**DOI:** 10.3390/life16071176

**Published:** 2026-07-16

**Authors:** Jirapat Wonglhow, Arunee Dechaphunkul, Patrapim Sunpaweravong, Chirawadee Sathitruangsak, Panu Wetwittayakhlang

**Affiliations:** 1 Division of Medical Oncology, Department of Internal Medicine, Faculty of Medicine, Prince of Songkla University, Songkhla 90110, Thailand; jirapat.jw@gmail.com (J.W.); dr.arunee@gmail.com (A.D.); spatrapi@medicine.psu.ac.th (P.S.); sjirawadee@gmail.com (C.S.); 2 Gastroenterology and Hepatology Unit, Division of Internal Medicine, Faculty of Medicine, Prince of Songkla University, Songkhla 90110, Thailand

**Keywords:** biliary tract cancer, cholangiocarcinoma, gemcitabine, palliative chemotherapy, overall survival, prognostic factors, alkaline phosphatase, neutrophil-to-lymphocyte ratio, performance status, risk stratification

## Abstract

Background: Although gemcitabine-based palliative chemotherapy remains widely used for advanced biliary tract cancer (BTC), practical pretreatment prognostic factors are needed. This study identified baseline prognostic factors associated with overall survival (OS) and explored a simple risk stratification approach for 12-month survival in advanced BTC patients treated with gemcitabine-based chemotherapy. Methods: This retrospective cohort study included advanced BTC patients treated with gemcitabine-based palliative chemotherapy between 2011 and 2025. Baseline clinical and laboratory variables were collected at treatment initiation. The Kaplan–Meier method estimated OS. Univariable and multivariable Cox proportional hazards regression analyses identified prognostic factors. A post hoc exploratory risk score was developed using routinely available factors independently associated with OS. Results: A total of 154 patients were included, gemcitabine plus cisplatin was administered to 95 patients, whereas 59 received gemcitabine plus carboplatin. Median OS was 9.43 months. Multivariable analysis showed that ECOG performance status ≥ 2 (adjusted HR, 5.68; 95% CI, 2.52–12.81), alkaline phosphatase (ALP) ≥ 2 × ULN (adjusted HR, 1.53; 95% CI, 1.01–2.33), and neutrophil-to-lymphocyte ratio (NLR) ≥ 3 (adjusted HR, 1.51; 95% CI, 1.03–2.20) were associated with worse OS. An exploratory score assigning one point to each factor stratified patients into low-, intermediate-, and high-risk groups. The estimated 12-month OS rates were 59.6%, 40.4%, and 10.8%, respectively. Conclusions: Poor performance status, elevated ALP, and elevated NLR were associated with worse OS in advanced BTC patients receiving gemcitabine-based palliative chemotherapy. However, the associations for ALP and NLR were modest and should be interpreted cautiously. A simple exploratory score based on routinely available factors demonstrated distinct 12-month survival across risk groups and may help inform prognostic discussions in routine practice. This approach should be considered hypothesis-generating, and external validation is warranted.

## 1. Introduction

Advanced biliary tract cancer (BTC) remains a clinically challenging malignancy with limited long-term survival despite advances in systemic therapy [1]. BTC includes intrahepatic cholangiocarcinoma, extrahepatic cholangiocarcinoma, and gallbladder cancer. Although these subtypes differ in anatomical origin, biological behavior, molecular profile, and clinical outcome, they are managed within a shared framework in advanced disease because of their rarity and similar therapeutic approach [2,3,4]. Many patients present with unresectable or metastatic disease, making systemic therapy the primary treatment modality for disease control and survival prolongation [5].

Gemcitabine-based chemotherapy has been a cornerstone of first-line treatment for advanced BTC for more than a decade. The phase III ABC-02 trial established gemcitabine plus cisplatin as the standard regimen by demonstrating a survival advantage over gemcitabine alone [6]. More recently, adding immunotherapy to gemcitabine and cisplatin has improved outcomes and changed the treatment landscape [7,8,9]. Nevertheless, gemcitabine-based palliative chemotherapy remains important in real-world practice [10,11,12]. A substantial proportion of patients may still receive chemotherapy alone because of contraindications to immunotherapy, limited access or reimbursement, financial constraints, or clinical factors that preclude chemoimmunotherapy.

Despite advances in systemic therapy, survival outcomes in advanced BTC remain highly variable. Some patients achieve meaningful disease control with chemotherapy, whereas others experience rapid clinical deterioration and early mortality. This heterogeneity reflects tumor burden, disease extent, and host-related factors such as performance status, hepatic function, nutritional reserve, and systemic inflammation [13,14,15,16]. However, the prognostic relevance of these factors may vary across patient populations, treatment patterns, and healthcare settings. In clinical practice, simple and readily available parameters are needed to support prognostic assessment before palliative chemotherapy.

Real-world data are important because patients in routine oncology practice are more heterogeneous than those in clinical trials. Older patients, those with impaired renal function, suboptimal performance status, biliary drainage, or comorbidities are often underrepresented in pivotal studies yet remain common in daily practice [10,12,17]. Identifying practical prognostic factors in patients receiving gemcitabine-based palliative chemotherapy may support risk stratification, patient counseling, treatment planning, early integration of supportive care, and supporting individualized decision-making. This study aimed to identify baseline clinical and laboratory prognostic factors associated with overall survival (OS) in patients with advanced BTC treated with gemcitabine-based palliative chemotherapy in a real-world setting. We also explored whether a simple risk stratification approach using routinely available prognostic domains could identify patients at high risk of poor 12-month survival.

## 2. Materials and Methods

### 2.1. Study Participants

This retrospective cohort study included patients with advanced BTC who received gemcitabine-based palliative chemotherapy at Songklanagarind Hospital, Prince of Songkla University between January 2011 and December 2025. Eligible patients were aged ≥18 years and had histologically confirmed adenocarcinoma BTC, including intrahepatic cholangiocarcinoma, extrahepatic cholangiocarcinoma, gallbladder cancer, or ampulla of Vater cancer. Patients included were those with unresectable locally advanced, recurrent, or metastatic disease who received at least one cycle of palliative gemcitabine-based chemotherapy. Patients who received chemotherapy only in the adjuvant or perioperative setting were excluded.

The study was conducted in accordance with the Declaration of Helsinki, and approved by the Human Research Ethics Committee, Faculty of Medicine, Prince of Songkla University (REC.69023141). Patient consent was waived due to the retrospective nature of the study. Patient confidentiality was maintained by anonymizing all identifiable information before analysis.

### 2.2. Treatment and Study Variables

Gemcitabine-based palliative chemotherapy was defined as gemcitabine administered in combination with a platinum agent, including cisplatin or carboplatin. The treatment regimen was selected by the treating physician based on clinical judgment, renal function, performance status, comorbidities, and expected treatment tolerance.

Baseline clinical and laboratory variables were collected at initiation of palliative chemotherapy. Clinical variables included age, sex, Eastern Cooperative Oncology Group (ECOG) performance status, body mass index, primary tumor site, disease extent, creatinine clearance, and chemotherapy regimen. Laboratory variables included complete blood count, liver function tests, renal function tests, and cancer antigen 19-9 (CA 19-9). The neutrophil-to-lymphocyte ratio (NLR) was derived from baseline complete blood count data.

### 2.3. Outcomes

The primary outcome was OS, defined as the time from initiation of gemcitabine-based palliative chemotherapy to death from any cause. Patients alive at the last follow-up were censored on the date of last contact. The main objective was to identify baseline clinical and laboratory factors associated with OS. Twelve-month OS was assessed using Kaplan–Meier estimates. Twelve-month OS was also used for exploratory risk stratification to identify patients at high risk of early mortality after initiation of palliative chemotherapy.

### 2.4. Statistical Analyses

Descriptive statistics were used to summarize baseline characteristics, treatment variables, and laboratory parameters. Categorical variables were presented as frequencies and percentages. Continuous variables were presented as medians with interquartile ranges (IQR) or means with standard deviations, as appropriate.

OS was estimated using the Kaplan–Meier method, and median OS was reported with 95% confidence intervals (CIs). Survival differences between groups were compared using the log-rank test. Candidate prognostic factors were selected a priori based on literature review, prior studies, and investigator clinical expertise. These factors included demographic characteristics, performance status, nutritional status, disease-related variables, renal and liver function parameters, inflammatory markers, tumor marker level, and chemotherapy regimen.

Univariable Cox proportional hazards regression assessed the association between each candidate prognostic factor and OS. Variables associated with OS at *p* < 0.05 in univariable analyses were entered into the multivariable Cox proportional hazards model. No automated stepwise selection procedure was used. Model complexity was assessed using the events-per-variable principle, with at least 10 events per covariate considered acceptable. Hazard ratios with 95% CIs were reported.

A post hoc exploratory risk stratification analysis was performed using baseline factors independently associated with OS in multivariable Cox regression analysis. Patients were categorized into risk groups based on a derived risk score. Twelve-month OS for each risk group was estimated using the Kaplan–Meier method. Continuous variables were categorized using clinically established criteria, laboratory reference limits, or cut-offs supported by prior prognostic literature [13]. Specifically, alkaline phosphatase (ALP) and total bilirubin were categorized relative to the upper limit of normal (ULN), while NLR ≥ 3 and CA 19-9 ≥ 120 U/mL were selected based on prior prognostic studies in BTC [13]. No automated cut-point optimization method was used. Because complete data were available for all variables included in the multivariable analysis and exploratory risk stratification, no data imputation was required. The apparent discriminatory performance of the exploratory risk score was assessed using Harrell’s concordance index (C-index), with the corresponding 95% CI. A two-sided *p*-value < 0.05 was considered statistically significant. Statistical analyses were performed using R software version 4.6.0 (R Foundation for Statistical Computing, Vienna, Austria).

## 3. Results

### 3.1. Patient Characteristics

A total of 154 patients with advanced BTC receiving gemcitabine-based palliative chemotherapy were included. The mean age was 60.4 years, and 50.6% of patients were male. Most patients had ECOG performance status 0–1 (92.2%) and metastatic disease at treatment initiation (92.8%). The most common primary tumor site was intrahepatic cholangiocarcinoma (50.6%), followed by extrahepatic cholangiocarcinoma (29.9%) and gallbladder cancer (14.3%). Gemcitabine plus cisplatin was administered to 95 patients (61.7%), whereas 59 patients (38.3%) received gemcitabine plus carboplatin. Further baseline clinical and laboratory findings are summarized in Table 1. Treatment details are presented in Table S1.

### 3.2. Survival Outcomes

**Figure 1:**
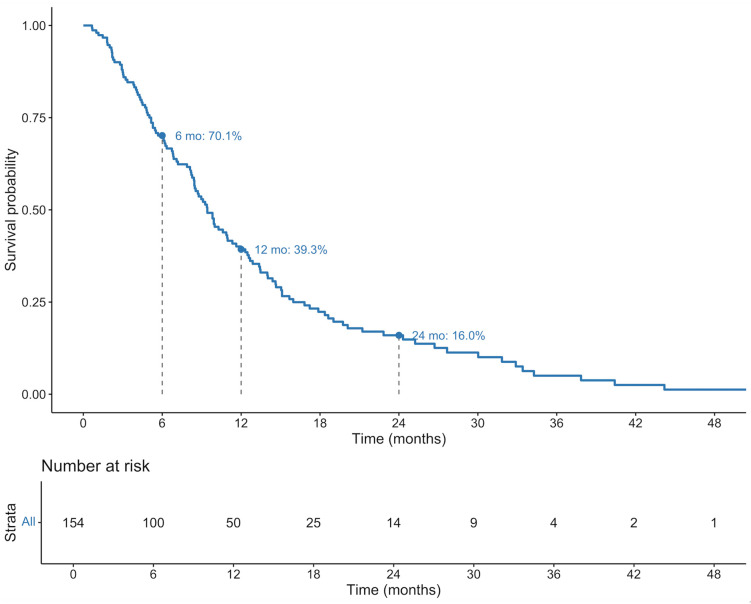
Kaplan–Meier curve for overall survival.

At the time of analysis, 127 patients (82.5%) had died, and 27 (17.5%) were censored at the last follow-up. The median observation time for the overall cohort, defined as the time from initiation of gemcitabine-based palliative chemotherapy to death or last follow-up, was 8.5 months (IQR, 4.5–14.3 months). The median OS for the overall cohort was 9.43 months (95% CI, 8.44–11.30). Estimated OS rates at 6, 12, and 24 months were 70.2%, 39.3%, and 16.0%, respectively (Figure 1). The median progression-free survival was 4.99 months (95% CI, 3.98–5.98). Response evaluations were available for 134 patients (87.0%), the objective response rate was 9.1% in the overall treatment population and 10.4% among patients with evaluable response data.

### 3.3. Prognostic Factors Associated with Overall Survival

Univariable and multivariable Cox regression analyses for OS are presented in Table 2. In univariable analysis, ECOG performance status ≥ 2, hypoalbuminemia, elevated CA 19-9, elevated ALP, elevated total bilirubin, metastatic disease, lung metastasis, elevated NLR, and leukocytosis were associated with worse OS.

In multivariable analysis, ECOG performance status ≥ 2 showed the strongest associated with inferior OS. Elevated ALP (≥2 × ULN) and NLR ≥ 3 also remained associated with worse OS after adjustment. Other variables were not independently associated with OS after adjustment. The multivariable Cox model included nine covariates. With 127 death events, the events-per-variable ratio was 14.1.

### 3.4. Exploratory Risk Stratification for 12-Month Overall Survival

**Figure 2:**
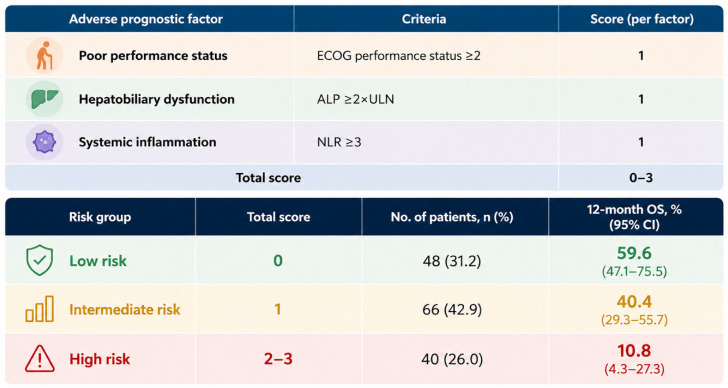
Exploratory risk stratification score for 12-month overall survival. ECOG, Eastern Cooperative Oncology Group; ALP, alkaline phosphatase; NLR, neutrophil-to-lymphocyte ratio; ULN, upper limit of normal.

Based on independently associated and routinely available baseline prognostic factors from multivariable analysis, an exploratory risk score was constructed using ECOG performance status ≥ 2, ALP ≥ 2 × ULN, and NLR ≥ 3. One point was assigned for each adverse factor, yielding a total score ranging from 0 to 3 (Figure 2).

**Figure 3:**
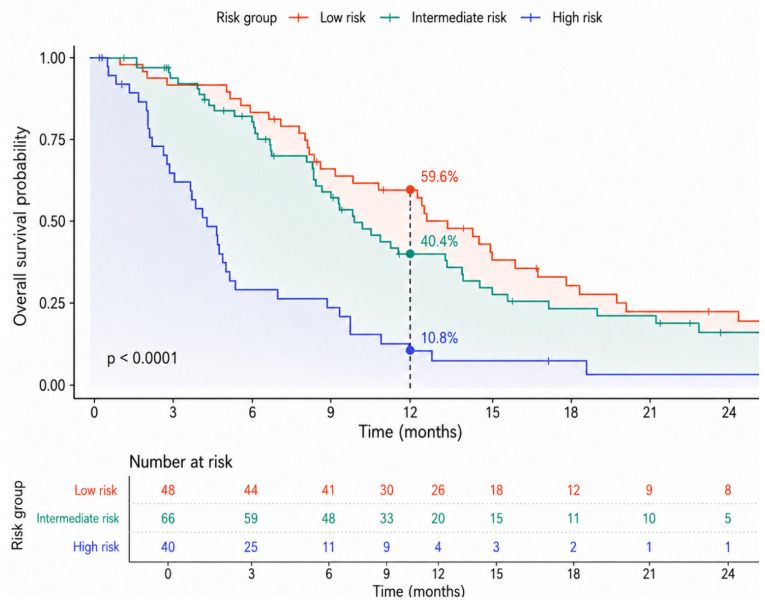
Kaplan–Meier curves for overall survival according to exploratory risk group (log-rank *p* < 0.0001).

The score distribution included 48 patients with a score of 0, 66 with a score of 1, 34 with a score of 2, and 6 with a score of 3. Accordingly, patients were categorized as low risk (score 0; *n* = 48), intermediate risk (score 1; *n* = 66), and high risk (scores 2–3; *n* = 40). Estimated 12-month OS rates showed a stepwise decrease across the low-, intermediate-, and high-risk groups: 59.6%, 40.4%, and 10.8%, respectively. Kaplan–Meier curves according to exploratory risk group are shown in Figure 3. The exploratory risk score demonstrated an apparent Harrell’s C-index of 0.648 (95% CI, 0.594–0.701), indicating modest discrimination within this cohort. However, this analysis was exploratory and descriptive, and was not intended to establish a validated prognostic prediction model.

## 4. Discussion

In this real-world cohort of patients with advanced BTC receiving gemcitabine-based palliative chemotherapy, poor performance status, elevated ALP, and elevated NLR were associated with inferior OS. These factors represent three practical prognostic domains: host functional reserve, hepatobiliary dysfunction or cholestatic tumor burden, and systemic inflammation. An exploratory risk stratification score based on these routinely available parameters distinguished stratified patients into groups with different 12-month survival outcomes. These findings support the value of simple baseline clinical assessment for estimating prognosis before starting palliative chemotherapy.

The median OS of 9.43 months in our cohort is consistent with the outcomes reported for patients receiving gemcitabine-based chemotherapy in routine practice [6,7,8,18,19,20,21]. Although pivotal trials have established gemcitabine-based chemotherapy and more recently chemoimmunotherapy as standard first-line treatments, their populations are typically selected according to performance status, organ function, and protocol-defined eligibility criteria. Real-world cohorts remain important because they include patients with broader clinical heterogeneity, including renal impairment, biliary obstruction, comorbidities, and variable treatment tolerance [14,19,22,23]. Therefore, prognostic information from routine practice remains clinically relevant, particularly when treatment decisions must balance expected benefit, toxicity, and patient condition.

Performance status was the strongest prognostic factor. Patients with ECOG performance status ≥ 2 had substantially worse survival, consistent with previous studies in advanced BTC [14,19,24,25]. This likely reflects reduced physiological reserve, higher symptom burden, and lower tolerance of systemic therapy. Elevated ALP was also associated with inferior OS and may indicate cholestasis, biliary obstruction, hepatic involvement, or extensive hepatobiliary tumor burden. Previous prognostic models in advanced BTC have identified liver involvement, biliary obstruction, albumin, inflammatory markers, and tumor markers as clinically relevant variables [13,16,26,27]. Although bilirubin was associated with survival in univariable analysis, ALP remained more consistent after adjustment, possibly because bilirubin is influenced by reversible obstruction or biliary drainage, whereas ALP may reflect cholestatic disease burden or liver involvement.

Elevated NLR, a marker of systemic inflammation, was another adverse prognostic factor. NLR is an inexpensive and readily available marker derived from routine complete blood count testing. A high NLR may reflect a pro-tumor inflammatory state, impaired host immune response, or aggressive tumor biology [28]. Previous reports have also shown that elevated NLR is associated with worse survival in BTC [29,30]. Our findings supported the use of NLR as an additional prognostic marker when interpreted alongside performance status and hepatobiliary function.

A secondary exploratory analysis was performed to describe survival heterogeneity according to routinely available baseline factors associated with OS. Although prognostic scores and nomograms for advanced BTC have previously been proposed based on clinical and laboratory factors [13,16,26,27], the present study explored a simpler risk stratification approach using ECOG performance status ≥ 2, ALP ≥ 2 × ULN, and NLR ≥ 3. This exploratory grouping demonstrated a stepwise decrease in 12-month OS across low-, intermediate-, and high-risk groups. However, the proposed grouping should not be interpreted as a validated prognostic model or as a basis for withholding chemotherapy. Rather, it may help illustrate how combinations of poor performance status, elevated ALP, and elevated NLR identify patients with markedly different survival outcomes in this cohort. These findings may help inform treatment discussions by highlighting patients who may require careful assessment of expected treatment benefit, toxicity risk, symptom burden, goals of care, early supportive care integration, and closer monitoring. Because all components are routinely available in clinical practice, this exploratory framework may be feasible for further evaluation. Nevertheless, it should be considered hypothesis-generating and requires validation in independent cohorts.

This study has several limitations. First, its retrospective, single-center design may introduce selection bias and unmeasured confounding. Treatment selection and dose modifications were at the discretion of the treating physicians. Second, the exploratory risk stratification was derived and assessed within the same cohort and was not designed as a formal prognostic prediction model. Although the apparent discrimination of the exploratory risk score was assessed using Harrell’s C-index, calibration and optimism-corrected performance were not evaluated. Because the score was derived and assessed within the same cohort, its discriminatory performance may be optimistic and requires confirmation in independent cohorts. The observed separation of survival curves should be interpreted as descriptive and hypothesis-generating rather than evidence of validated predictive utility. In addition, the associations between ALP ≥ 2 × ULN and NLR ≥ 3 were modest, with 95% CIs close to 1.0, and should therefore be interpreted cautiously. External validation in an independent cohort is required before this exploratory approach can be applied in clinical practice. Finally, the findings may not be directly generalizable to patients receiving contemporary chemoimmunotherapy regimens.

In conclusion, poor performance status, elevated ALP, and elevated NLR were associated with worse OS in patients with advanced BTC receiving gemcitabine-based palliative chemotherapy. A simple exploratory risk stratification based on these routinely available factors demonstrated the different 12-month survival outcome across groups. However, this approach should be interpreted as hypothesis-generating rather than a validated prognostic model, and external validation is warranted.

## Figures and Tables

**Table 1:** Patient characteristics.

Characteristics	Total (*n* = 154)
Mean age, years (SD)	60.4 (10.3)
Sex, *n* (%)	
Male	78 (50.6)
Female	76 (49.4)
ECOG PS, *n* (%)	
0–1	142 (92.2)
≥2	12 (7.8)
BMI, *n* (%)	
<18.5 kg/m^2^	35 (22.7)
18.5–22.9 kg/m^2^	67 (43.5)
≥23.0 kg/m^2^	52 (33.8)
Cirrhosis, *n* (%)	10 (6.5)
Laboratory values	
Hemoglobin, g/dL (SD)	11.5 (1.60)
White blood cell, /μL (IQR)	8470 (6880, 10,630)
White blood cell ≥ 10,000/μL, *n* (%)	51 (33.1)
NLR (IQR)	3.46 (2.02, 5.79)
NLR ≥ 3, *n* (%)	87 (56.5)
Platelet, /μL (IQR)	298,000 (243,000, 357,750)
Total bilirubin, mg/dL (IQR)	0.7 (0.4, 1.1)
Total bilirubin ≥ ULN, *n* (%)	30 (19.5)
AST, U/L (IQR)	37 (24.0, 57.0)
ALT, U/L (IQR)	23 (17.0, 45.8)
ALP, U/L (IQR)	160 (112.5, 318.8)
ALP ≥ 2 × ULN, *n* (%)	53 (34.4)
Albumin, g/dL (IQR)	3.9 (3.4, 4.3)
Albumin < 3.5 g/dL, *n* (%)	43 (27.9)
CA 19-9, U/mL (IQR)	85.1 (10.0, 1393.8)
CA 19-9 ≥ 120 U/mL, *n* (%)	71 (46.1)
Creatinine clearance, mL/min (IQR)	55.1 (44.7, 69.5)
Primary tumor site, *n* (%)	
Intrahepatic bile duct	78 (50.6)
Extrahepatic bile duct	46 (29.9)
Gallbladder	22 (14.3)
Ampulla of Vater	8 (5.2)
Advanced disease status, *n* (%)	
Unresectable locally advanced disease	11 (7.1)
Metastatic disease	143 (92.8)
Number of organ metastasis, *n* (%)	
1	79 (51.3)
2	40 (26.0)
≥3	24 (15.6)
Organ metastasis, *n* (%)	
Liver	78 (50.6)
Lung	38 (24.7)
Lymph nodes	72 (46.8)
Peritoneum	33 (21.4)
Bone	14 (9.1)
Regimen, *n* (%)	
Gemcitabine plus cisplatin	95 (61.7)
Gemcitabine plus carboplatin	59 (38.3)

ECOG PS, Eastern Cooperative Oncology Group performance status; BMI, body mass index; NLR, neutrophil-to-lymphocyte ratio; AST, aspartate aminotransferase; ALT, alanine aminotransferase; ALP, alkaline phosphatase; CA 19-9, cancer antigen 19-9.

**Table 2:** Prognostic factors for overall survival.

	Univariable Analysis		Multivariable Analysis	
	HR (95% CI)	*p* Value	HR (95% CI)	*p* Value
Age ≥ 65 years	1.02 (0.69, 1.52)	0.917	-	-
Male	1.05 (0.74, 1.50)	0.770	-	-
BMI < 18.5 kg/m^2^	0.92 (0.61, 1.39)	0.688	-	-
ECOG PS ≥ 2	7.48 (3.57, 15.7)	<0.001	5.68 (2.52, 12.81)	<0.001
Albumin < 3.5 g/dL	2.64 (1.77, 3.96)	<0.001	1.40 (0.85, 2.30)	0.186
CA 19-9 ≥ 120 U/mL	1.43 (1.01, 2.03)	0.046	1.12 (0.75, 1.68)	0.574
ALP ≥ 2 × ULN	2.04 (1.41, 2.95)	<0.001	1.53 (1.01, 2.33)	0.044
TB > ULN	1.81 (1.15, 2.83)	0.010	1.57 (0.92, 2.68)	0.097
CrCl < 60 mL/min	0.90 (0.63, 1.28)	0.556	-	-
NLR ≥ 3	1.72 (1.21, 2.46)	0.003	1.51 (1.03, 2.20)	0.034
Hb < 10 g/dL	1.54 (0.98, 2.43)	0.062	-	-
WBC > 10,000/μL	1.94 (1.34, 2.81)	<0.001	1.31 (0.87, 1.97)	0.199
Plt < 150,000/μL	1.01 (0.44, 2.31)	0.976	-	-
Primary tumor site			-	-
Intrahepatic bile duct	Ref	Ref		
Extrahepatic bile duct	1.00 (0.67, 1.50)	0.998		
Gallbladder	1.16 (0.68, 1.97)	0.580		
Ampulla of Vater	0.49 (0.21, 1.14)	0.097		
Metastatic vs. locally advanced disease	2.11 (1.06, 4.22)	0.035	1.65 (0.81, 3.37)	0.171
Lung metastasis	1.53 (1.02, 2.29)	0.039	1.44 (0.91, 2.27)	0.116
Liver metastasis	1.12 (0.79, 1.60)	0.526	-	-
Peritoneal metastasis	1.15 (0.74, 1.78)	0.538	-	-
Cisplatin vs. carboplatin	0.85 (0.59, 1.22)	0.382	-	-

ECOG PS, Eastern Cooperative Oncology Group performance status; BMI, body mass index; NLR, neutrophil-to-lymphocyte ratio; ALP, alkaline phosphatase; CA 19-9, cancer antigen 19-9; CrCl, creatinine clearance; Hb, hemoglobin; WBC, white blood cell; Plt, platelet; TB, total bilirubin; ULN, upper limit of normal; HR, hazard ratio; CI, confidence interval.

## Data Availability

The datasets used and/or analyzed during the current study are available from the corresponding author upon request.

## Supplementary Materials

The following supporting information can be downloaded at: https://www.mdpi.com/article/10.3390/life16071176/s1, Table S1: treatment details.

## Abbreviations

The following abbreviations are used in this manuscript:

BTC	Biliary Tract Cancer
OS	Overall Survival
ECOG	Eastern Cooperative Oncology Group
ALP	Alkaline Phosphatase
ULN	Upper Limit of Normal
NLR	Neutrophil-to-Lymphocyte Ratio
CI	Confidence Interval
CA 19-9	Cancer Antigen 19-9
ABC-02	Advanced Biliary Cancer-02 (phase III clinical trial)

## References

[1] Bray F., Laversanne M., Sung H., Ferlay J., Siegel R.L., Soerjomataram I., Jemal A. (2024). Global cancer statistics 2022: GLOBOCAN estimates of incidence and mortality worldwide for 36 cancers in 185 countries. CA Cancer J. Clin..

[2] Vogel A., Ducreux M. (2025). ESMO Clinical Practice Guideline interim update on the management of biliary tract cancer. ESMO Open.

[3] Banales J.M., Marin J.J.G., Lamarca A., Rodrigues P.M., Khan S.A., Roberts L.R., Cardinale V., Carpino G., Andersen J.B., Braconi C. (2020). Cholangiocarcinoma 2020: The next horizon in mechanisms and management. Nat. Rev. Gastroenterol. Hepatol..

[4] Ustundag Y., Bayraktar Y. (2008). Cholangiocarcinoma: A compact review of the literature. World J. Gastroenterol..

[5] Benson A.B., D’Angelica M.I., Abrams T., Ahmed A., Akce M., Anaya D.A., Anders R., Are C., Aye L., Bachini M. (2025). Biliary tract cancers, Version 2.2025, NCCN Clinical Practice Guidelines In Oncology. J. Natl. Compr. Cancer Netw..

[6] Valle J., Wasan H., Palmer D.H., Cunningham D., Anthoney A., Maraveyas A., Madhusudan S., Iveson T., Hughes S., Pereira S.P. (2010). Cisplatin plus gemcitabine versus gemcitabine for biliary tract cancer. N. Engl. J. Med..

[7] Kelley R.K., Ueno M., Yoo C., Finn R.S., Furuse J., Ren Z., Yau T., Klümpen H.-J., Ozaka M., Verslype C. (2023). Pembrolizumab in combination with gemcitabine and cisplatin compared with gemcitabine and cisplatin alone for patients with advanced biliary tract cancer (KEYNOTE-966): A randomised, double-blind, placebo-controlled, phase 3 trial. Lancet.

[8] Oh D.-Y., He A.R., Bouattour M., Okusaka T., Qin S., Chen L.-T., Kitano M., Lee C.-K., Kim J.W., Chen M.-H. (2024). Durvalumab or placebo plus gemcitabine and cisplatin in participants with advanced biliary tract cancer (TOPAZ-1): Updated overall survival from a randomised phase 3 study. Lancet Gastroenterol. Hepatol..

[9] Oh D.-Y., He A.R., Qin S., Chen L.-T., Okusaka T., Vogel A., Kim J.W., Suksombooncharoen T., Lee M.A., Kitano M. (2022). Durvalumab plus gemcitabine and cisplatin in advanced biliary tract cancer. NEJM Evid..

[10] Seung S.J., Saherawala H., Syed I., Shephard C., Clouthier D.L., Chen E. (2023). Real-world treatment patterns and survival outcomes for treated biliary tract cancer patients using administrative databases in Ontario. J. Gastrointest. Oncol..

[11] Babu V.K., Talwar V., Raina S., Goel V., Dash P., Bajaj R., Sharma M., Medisetty P., Ram D., Agrawal C. (2018). Gemcitabine with carboplatin for advanced intrahepatic cholangiocarcinoma: A study from North India Cancer Centre. Indian J. Cancer.

[12] Maeda O., Ebata T., Shimokata T., Matsuoka A., Inada-Inoue M., Morita S., Takano Y., Urakawa H., Miyai Y., Sugishita M. (2020). Chemotherapy for biliary tract cancer: Real-world experience in a single institute. Nagoya J. Med. Sci..

[13] Park H.S., Park J.S., Chun Y.J., Roh Y.H., Moon J., Chon H.J., Choi H.J., Park J.S., Lee D.K., Lee S.-J. (2017). Prognostic factors and scoring model for survival in metastatic biliary tract cancer. Cancer Res. Treat..

[14] Suzuki Y., Kan M., Kimura G., Umemoto K., Watanabe K., Sasaki M., Takahashi H., Hashimoto Y., Imaoka H., Ohno I. (2019). Predictive factors of the treatment outcome in patients with advanced biliary tract cancer receiving gemcitabine plus cisplatin as first-line chemotherapy. J. Gastroenterol..

[15] Felismino T.C., Oliveira F.M.A., Fogassa C.A.Z., Santerini S.N., de Jesus V.H.F., Riechelmann R.S.P., Coimbra F.J.F., Mello C.A.L. (2022). Evaluation of prognostic factors in patients undergoing first-line chemotherapy for advanced biliary tract cancer: A retrospective analysis from a South American cancer centre. Ecancermedicalscience.

[16] Imaoka H., Ikeda M., Nomura S., Morizane C., Okusaka T., Ozaka M., Shimizu S., Yamazaki K., Okano N., Sugimori K. (2023). Development of a nomogram to predict survival in advanced biliary tract cancer. Sci. Rep..

[17] Baraka B., Ponda D.J., Hanna J., Gomez D., Aithal G., Arora A. (2025). Efficacy and toxicity profile of carboplatin/gemcitabine chemotherapy in locally advanced or metastatic biliary tract cancer: A single UK centre experience. Cancers.

[18] Lee J., Kim T.-Y., Lee M.A., Ahn M.J., Kim H.-K., Lim H.Y., Lee N.S., Park B.J., Kim J.S., On Behalf of the Korean Cancer Study Group (2008). Phase II trial of gemcitabine combined with cisplatin in patients with inoperable biliary tract carcinomas. Cancer Chemother. Pharmacol..

[19] Park J.O., Oh D.-Y., Hsu C., Chen J.-S., Chen L.-T., Orlando M., Kim J.S., Lim H.Y. (2015). Gemcitabine plus cisplatin for advanced biliary tract cancer: A systematic review. Cancer Res. Treat..

[20] Thongprasert S., Napapan S., Charoentum C., Moonprakan S. (2005). Phase II study of gemcitabine and cisplatin as first-line chemotherapy in inoperable biliary tract carcinoma. Ann. Oncol..

[21] Wonglhow J., Wong H.-L., Michael M. (2025). Effectiveness of chemotherapy plus immunotherapy and molecular alterations in advanced biliary tract cancer: Real-world evidence from a single-center Australian cohort. Cancer Control.

[22] Wu C.-E., Chou W.-C., Hsieh C.-H., Chang J.W.-C., Lin C.-Y., Yeh C.-N., Chen J.S. (2020). Prognostic and predictive factors for Taiwanese patients with advanced biliary tract cancer undergoing frontline chemotherapy with gemcitabine and cisplatin: A real-world experience. BMC Cancer.

[23] Agarwal R., Sendilnathan A., Siddiqi N.I., Gulati S., Ghose A., Xie C., Olowokure O.O. (2016). Advanced biliary tract cancer: Clinical outcomes with ABC-02 regimen and analysis of prognostic factors in a tertiary care center in the United States. J. Gastrointest. Oncol..

[24] Suzuki E., Furuse J., Ikeda M., Okusaka T., Nakachi K., Mitsunaga S., Ueno H., Morizane C., Kondo S., Shimizu S. (2010). Treatment efficacy/safety and prognostic factors in patients with advanced biliary tract cancer receiving gemcitabine monotherapy: An analysis of 100 cases. Oncology.

[25] Saisho T., Okusaka T., Ueno H., Morizane C., Okada S. (2005). Prognostic factors in patients with advanced biliary tract cancer receiving chemotherapy. Hepatogastroenterology.

[26] Salati M., Caputo F., Cunningham D., Marcheselli L., Spallanzani A., Rimini M., Gelsomino F., Reggiani-Bonetti L., Andrikou K., Rovinelli F. (2019). The A.L.A.N. score identifies prognostic classes in advanced biliary cancer patients receiving first-line chemotherapy. Eur. J. Cancer.

[27] Wu C.-E., Huang W.-K., Chou W.-C., Hsieh C.-H., Chang J.W.-C., Lin C.-Y., Yeh C.-N., Chen J.-S. (2021). Establishment of a pretreatment nomogram to predict the 6-month mortality rate of patients with advanced biliary tract cancers undergoing gemcitabine-based chemotherapy. Cancers.

[28] Buonacera A., Stancanelli B., Colaci M., Malatino L. (2022). Neutrophil to lymphocyte ratio: An emerging marker of the relationships between the immune system and diseases. Int. J. Mol. Sci..

[29] McNamara M.G., Templeton A.J., Maganti M., Walter T., Horgan A.M., McKeever L., Min T., Amir E., Knox J. (2014). Neutrophil/lymphocyte ratio as a prognostic factor in biliary tract cancer. Eur. J. Cancer.

[30] Tang H., Lu W., Li B., Li C., Xu Y., Dong J. (2017). Prognostic significance of neutrophil-to-lymphocyte ratio in biliary tract cancers: A systematic review and meta-analysis. Oncotarget.

