# Supplementary material for: Clinical Prognostic Factors and Survival Risk Stratification for Advanced Biliary Tract Cancer Treated with Gemcitabine-Based Palliative Chemotherapy: A Real-World Retrospective Study
Source: Life (Basel). 2026 Jul 16;16(7):1176. doi: 10.3390/life16071176 (PMC13413110; doi:10.3390/life16071176)
Supplement: Supplementary file 1 [file life-16-01176-s001.zip › life-4398226-supplementary.pdf]

Table S1 Treatment details

Treatment information	GemCis (n = 95)	GemCarbo (n = 59)
Number of cycles (IQR)	6 (3, 8)	4 (2, 6)
Initial dose reduction	26 (27.4)	28 (47.5)
Previous treatment, n (%)		
Surgery	20 (21.1)	19 (32.2)
Adjuvant chemotherapy	8 (8.4)	6 (10.2)
Biliary drainage	40 (42.1)	22 (37.3)
Discontinuation, n (%)		
Progressive disease	59 (62.1)	23 (39.0)
Complete treatment	13 (13.7)	13 (22.0)
PS deterioration	16 (16.8)	13 (22.1)
Infection	2 (2.1)	4 (6.8)
Death	3 (3.2)	1 (1.7)
Loss of follow-up	2 (2.1)	5 (8.5)
Subsequent treatment, n (%)		
Second-line chemotherapy	53 (55.8)	18 (30.5)
Third-line chemotherapy	20 (21.1)	4 (6.8)
